# β3GNT9 as a prognostic biomarker in glioblastoma and its association with glioblastoma immune infiltration, migration and invasion

**DOI:** 10.3389/fonc.2023.1214413

**Published:** 2023-09-12

**Authors:** YingHao Luo, Kan Wang, Lu Zhan, Fanyue Zeng, Jie Zheng, Sijing Chen, Xingbang Duan, Donghui Ju

**Affiliations:** Department of Neurosurgery, The Fourth Affiliated Hospital of Harbin Medical University, Harbin, China

**Keywords:** glioblastoma, β3GNT9, immune infiltration, TCGA, prognostic biomarker

## Abstract

**Background:**

Studies have shown that the immune infiltration of tumor microenvironment is related to the prognosis of glioblastoma, which is characterized by high heterogeneity, high recurrence rate and low survival rate. To unravel the role of β1,3-N-acetylglucosaminyltransferase-9 (β3GNT9) in the progression of glioblastoma, this study identifies the value of β3GNT9 as a prognostic biomarker in glioblastoma, and investigates the relationship between β3GNT9 expression and glioblastoma immune infiltration, migration and invasion

**Methods:**

β3GNT9 expression in glioblastoma was analyzed using the GEPIA database. The clinical features of glioblastoma were screened out from the TCGA database. The relationship between β3GNT9 expression and clinical features was analyzed. The relationship between β3GNT9 and the prognosis of glioblastoma was evaluated through univariate and multivariate COX regression analyses, and the survival analysis was conducted using the Kaplan-Meier method. GSEA was employed to predict the signaling pathway of β3GNT9 in glioblastoma. The correlation between β3GNT9 and tumor immune infiltration was analyzed using the related modules of CIBERSORT and TIMER. A172, U87MG and U251 cell lines were selected to verify β3GNT9 expression *in vitro*. The effects of β3GNT9 on the migration and invasion of glioblastoma were investigated through cell scratch and invasion assays.

**Results:**

β3GNT9 expression in glioblastoma group was significantly higher than that in normal brain tissue group (*P*<0.05). The overall survival rate in high β3GNT9 expression group was significantly lower than that in low β3GNT9 expression group (*P*<0.05). Regression analyses suggested that β3GNT9, involved primarily in glucosamine degradation and extracellular matrix receptor interaction, could be an independent prognostic factor for glioblastoma. CIBERSORT and GEPIA database analyses showed that β3GNT9 was correlated with tumor infiltrating immune cells such as T follicular helper cells, activating natural killer cells, monocytes, macrophages, and eosinophils, thus affecting the immune microenvironment of glioblastoma. Cell experiments confirmed that β3GNT9 was highly expressed in A172, U87MG and U251 cell lines (*P*<0.05), and promoted the migration and invasion of glioblastoma (*P*<0.05).

**Conclusion:**

The increased expression of β3GNT9 in glioblastoma can affect the immune microenvironment of glioblastoma and promote its migration and invasion. β3GNT9 can be used as a potential independent prognostic biomarker for patients with glioblastoma.

## Introduction

Originating from glial cells, glioma is the most common primary malignancy in the central nervous system (CNS) (1). In China, the annual incidence of glioma is 5/100,000~8/100,000, and the 5-year case fatality rate is second only to pancreatic cancer and lung cancer (2). Among all gliomas, glioblastoma is the most malignant, which is characterized by high heterogeneity, high recurrence rate and low survival rate (3). Despite various advances in surgical resection, radiotherapy, and chemotherapy, the prognosis of glioblastoma patients remains poor (4). The current standard treatment for glioma includes the maximum safe surgical resection of the tumor, supplemented by postoperative radiotherapy and chemotherapy. However, the median survival time of patients is only 14.6 months (5, 6). Given the poor prognosis of glioblastoma patients, an increasing number of clinicians are studying the molecular markers associated with glioblastoma, purporting to identify biomarkers that can predict glioblastoma prognosis so as to develop individualized treatment plans and improve the survival rate of glioblastoma patients.

β1,3-N-acetylglucosaminyltransferase-9 (β3GNT9) is one of the members of the β3-N-acetylglucosaminyltransferase (β3GlcNAcT) family (7). As a type 2 transmembrane protein (7), the β1-3-N-acetylglucosaminyltransferases (β3GNTs) family is found to be associated with a variety of malignant tumor cells, such as lung adenocarcinoma (8), cervical cancer (9), triple-negative breast cancer (10), and so forth. In recent years, studies have shown that the main molecular biological function of β3GNTs is to regulate the activity of glycosyltransferases, mainly through participating in biological processes such as protein glycosylation, and then affecting the metabolism and growth of tumor cells (11). Tumors are organ-like tissues whose development is a result of the co-evolution of tumor cells and their surroundings (12). A more recent and groundbreaking idea is that cancer is not a genetic disease but an ecological disease: a multidimensional spatiotemporal “unity of ecology and evolution” pathological ecosystem (13). The cell environment in which the tumor cells live is called the tumor microenvironment, including stromal cells, immune-infiltrating cells, extracellular matrix, and so forth (14). As a major component of the tumor microenvironment, immune-infiltrating cells have drawn increasing scholarly interest. However, to date there is no report on the expression of β3GNT9 in glioblastoma and its relationship with clinical prognosis and immune-infiltrating cells. To address this lacuna, the current study employed bioinformatics techniques to identify the value of β3GNT9 in glioblastoma, and explore the relationship between β3GNT9 expression and glioblastoma immune infiltration, migration and invasion.

## Materials and methods

### Data source

The dataset for this study consisted of the mRNA transcriptome data and the clinical information of 169 glioblastoma tissues, as well as the mRNA transcriptomic data and the clinical information of 207 normal brain tissues. The former was downloaded from The Cancer Genome Atlas (TCGA) database (https://portal.gdc.cancer.gov), while the latter from the Genotype-Tissue Expression (GTEx) database (https://commonfund.nih.gov/gtex).

### Differential gene screening

The 207 normal brain tissues were set as control group, and the 169 glioblastoma tissues were set as experimental group. The limma package in R was used to screen differential genes under the threshold of log2FC absolute value > 1 and a *P* value < 0.05 (15).

### β3GNT9 expression and glioblastoma prognosis analysis

The Gene Expression Profiling Interactive Analysis (GEPIA) database is a web tool based on the TCGA and GTEx data for cancer and normal gene expression profiles as well as interactive analysis (16). We used the Expression module in the GEPIA database to analyze the differential expressions of β3GNT9 in normal tissues and in glioblastoma tissues. The median of β3GNT9 expression was taken as the reference point to divide the tissues into high and low expression groups, and the “survival” package in R was employed to draw the Kaplan-Meier survival curves to analyze the impact of β3GNT9 on the prognosis of glioblastoma patients. Later, based on the survival data of glioblastoma patients in the TCGA-GBM database combined with their β3GNT9 expression, univariate and multivariate COX regression analyses were conducted to figure out the effects of clinical factors and β3GNT9 on patient survival.

### Gene set enrichment analysis

Gene Set Enrichment Analysis (GSEA) is a bioinformatics analysis method used to determine whether there is such a gene set that consists of genes with biological function, chromosomal location, or regulation (17). GSEA can be applied to predict the potential working mechanism of the studied genes. In this study, gene enrichment analysis was performed using the GSEA 4.1.0 software. We included gene sets with a *P* value < 0.05 and false discovery rate (FDR) < 0.05 as significant enriched gene sets.

### Analysis of the immune infiltration

CIBERSORT (http://cibersort.stanford.edu/), a method for assessing the composition of tissue cells based on gene expression profiles, is a gene expression-based deconvolution algorithm that evaluates the expression changes of a set of genes relative to other genes in a sample (18). We assessed the immune response of 22 tumor immune-infiltrating cells in glioblastomas using CBIERSORT to identify the relationship between survival and molecular composition. We uploaded the data-standardized glioblastoma mRNA dataset to CIBERSORT, and obtained 22 immune tumor immune-infiltrating cells data when setting permutation displacement 1000 times. Glioblastoma patients were divided into screening gene high expression groups and low expression groups, and then the differences of immune cells between β3GNT9 high expression group and low expression group were identified and then visualized.

### TIMER database analysis

TIMER (https://cistrome.shinyapps.io/timer/) database, a web server for comprehensive analysis of tumor-infiltrating immune cells, covers 32 tumors with a total of 10897 samples (19). The TIMER database includes six modules: gene module, survival module, mutation module, somatic copy number module, and gene expression module. We used the gene module to analyze the correlation between β3GNT9 and the infiltration levels of six immune cells (i.e., B cells, CD8+T cells, CD4+T cells, macrophages, neutrophils and dendritic cells) in glioblastomas.

### Cell culture

The human astrocyte cell line NHA and the human glioblastoma cell lines A172, U87MG, U251 were purchased from Procell Life Science & Technology Company (Wuhan, China) and cultured in the Dulbecco’s Modified Eagle Medium (DMEM; Gibco, Grand Island, NY, USA) containing 10% fetal bovine serum (FBS; Gibco, Grand Island, NY, USA) and 1% penicillin/streptomycin (Cyagen Biosciences, Guangzhou, China) at 37°C in a cell incubator with an atmosphere of 5% CO_2_ and humidity saturation (20). The culture medium was replaced once every 2 days. Cells in the log growth phase were taken for the experiment.

### Cell transfection

When the cells reached 70% confluence, β3GNT9-shRNA was transfected into U251 cells informed by the procedure instruction of the transfection reagent Lipofectamine TM2000 (Invitrogen, Carlsbad, CA, USA) (21). The β3GNT9 knockdown shRNA plasmid was purchased from Shanghai Genechem Co., Ltd. (Shanghai, China).

### Quantitative real-time PCR

Four logarithmically growing cell lines were taken and RNA was extracted according to the Trizol instructions (Toyobo (Shanghai) Biotech, Shanghai, China). Reverse transcription reactions were performed according to the instructions by steps on the test kit (Toyobo (Shanghai) Biotech, Shanghai, China) and the quantitative real-time PCR according to the manufacturer’s instructions (Roche, Basel Switzerland). The primer sequences of β3GNT9 (forward: 5′-GCTCAAGGAGATCCACTTTCT-3′, reverse: 5′-CAGGAGATTTCCCACGTTCA-3′) and the primer sequences of the internal reference GAPDH (forward: 5′-CTGGGCTACACTGAGCACC-3′, reverse: 5′-AAGTGGTCGTTGAGGGCAATG-3′) were used. The expression levels of β3GNT9 mRNA in the cells were analyzed through the 2-ΔΔCt assay (22).

### Cell scratch test

At 6 hours post-transfection, the cells were digested, centrifuged, and re-suspended in FBS-free cultures. The cell concentration was adjusted to 2×10^5^ cells/ml. After cell mounting, the cell layer was then scratched with a 200 μl of sterile pipette tip. After removing cell culture medium and suspended cells and cell debris, each well was added into serum-free medium and cultured in the incubator for 24 and 48 hours when cell migration areas were photographed. The scratch assays were evaluated for differences in terms of the healing ability.

### Cell invasion assay

Matrigel (Corning, NY, USA) was melted at 4°C overnight. Then 100 μl of diluted matrix gel was added to the chamber. After that, 200 μl of serum-free medium was added to the upper chamber, and 500 μl of DMEM containing 10% FBS was added to the lower chamber. The collected cells with a density of 2×10^5^ cells/ml were planted in the upper chamber of culture plate and cultured in the incubator for an additional 48 hours. Subsequently, the invasion chamber was removed, and the cells on the polycarbonate membranes were fixed with 4% paraformaldehyde (Beyotime, Shanghai, China), and then stained with 0.1% crystal violet (Beyotime, Shanghai, China). Three fields were selected at random, and the invading cells were counted under the microscope.

### Statistical analysis

We utilized R programming language (version 3.6.3; https://www.r-project.org) and GraphPad Prism 7 (GraphPad Software, San Diego, CA, USA) for statistical analysis. The research data are presented as the means ± standard error of the means (SEM) or standard deviation (SD). A *P* value less than 0.05 was considered significant. The comparison of the experimental data was evaluated using the Student’s t test for two groups and one-way analysis of variance (ANOVA) for multiple groups. Differences in β3GNT9 expression between normal tissues and tumor tissues were analyzed using Wilcoxon signed-rank tests and one-way analysis of variance. The association between β3GNT9 expression and clinicopathological features was analyzed using the Kruskal-Wallis test, Dunn’s test, and Wilcoxon rank-sum test. Moreover, the evaluation of the prognostic significance of β3GNT9 expression was based on Kaplan-Meier method and Cox regression analysis. Independent prognostic factors for glioblastoma were identified via the R package. Univariate and multivariate Cox proportional hazard regression analyses were conducted to exclude β3GNT9 and four clinicodemographic characteristics (age, sex, radiation and pharmaceutical) from overall survival (OS) analysis.

## Results

### The screening results of the differentially expressed genes

**Figure 1:**
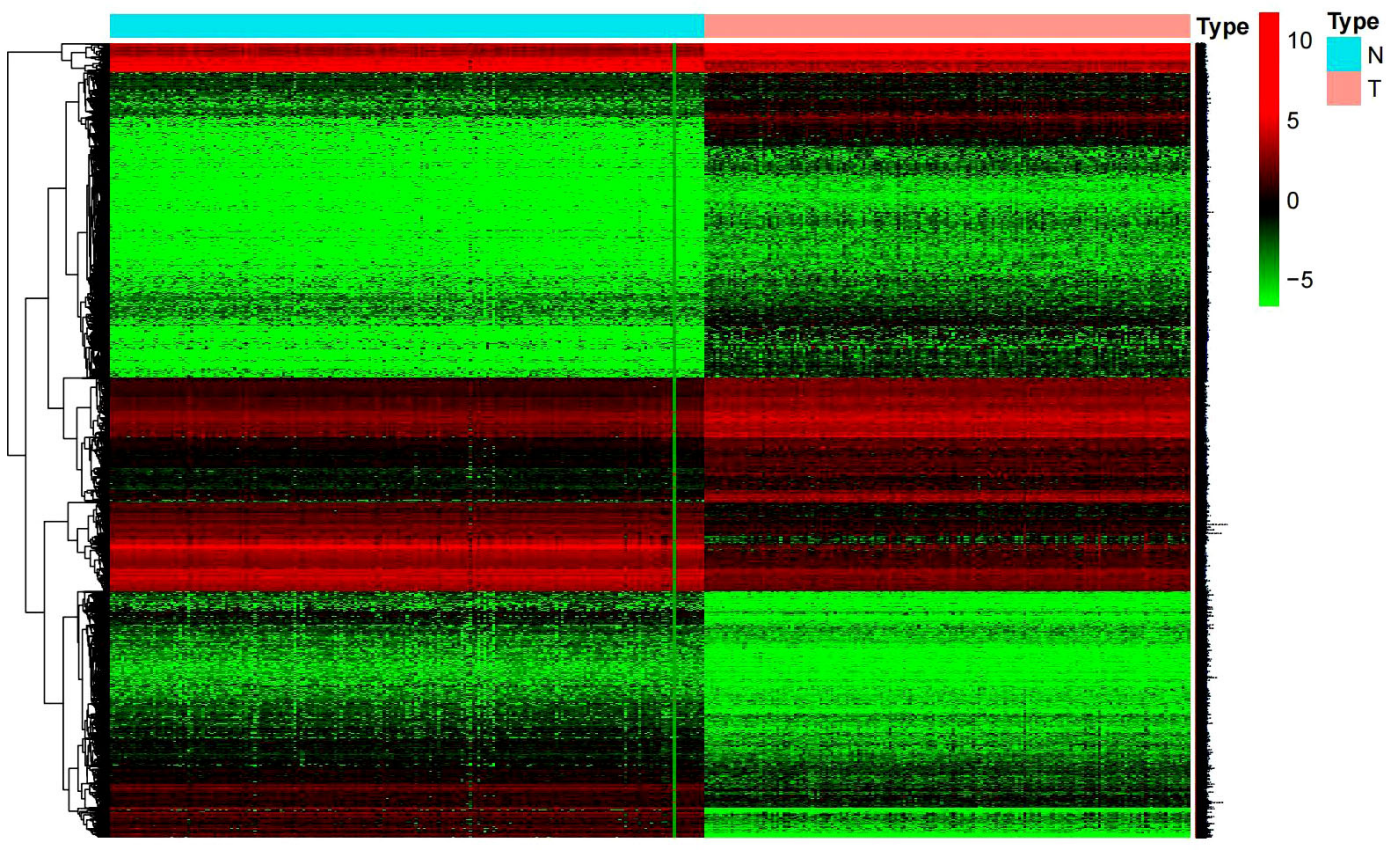
Heat map of differential genes. (Normal tissue in blue, glioblastoma tumor tissue in red).

Differential genes were screened using the limma package under the threshold of log2FC absolute value > 1 and a *P* value < 0.05. 18689 differential genes were obtained with their heatmap shown in **Figure 1**. These differential genes displayed different levels of expression only in tumor and normal tissues. Prognostic genes were obtained when further combining the clinical survival data with differential genes. We searched for those genes that have high clinical correlation but had not been studied previously, and finally we identified β3GNT9.

### β3GNT9 expression was significantly upregulated in glioblastoma tissues

**Figure 2:**
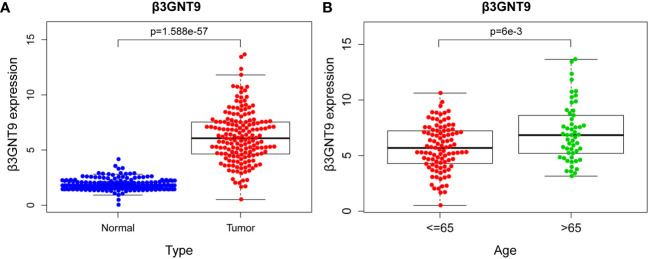
The results of β3GNT9 expression in glioblastoma. **(A)** β3GNT9 expression was higher in glioblastoma tissues than in normal tissues (*P*<0.05). **(B)** β3GNT9 expression was higher in patients older than 65 than in those of or younger than 65 (*P*<0.05).

Difference analysis was conducted through combining TCGA-GBM and GTEx data. The results showed that the expression of β3GNT9 in glioblastoma tissues was significantly higher than that in normal tissues (*P*<0.05, **Figure 2A**). Further exploration of the relationship between β3GNT9 and clinically relevant indicators of glioblastoma suggested that patients older than 65 years of age had higher β3GNT9 expression than patients of or under 65 years of age (*P*<0.05, **Figure 2B**).

### Prognostic value of β3GNT9 in glioblastoma

**Figure 3:**
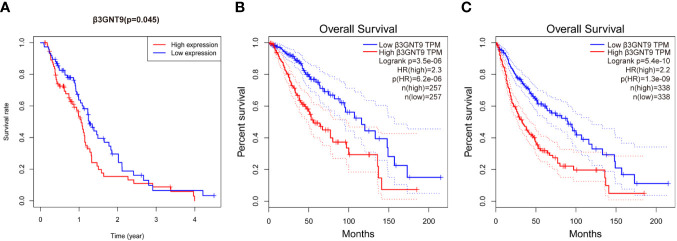
Prognostic value of β3GNT9 in glioblastoma. Kaplan-Meier survival curve shows increased expression of β3GNT9 was associated with poor OS in **(A)** glioblastoma patients (*P*<0.05); **(B)** low-grade glioma patients (*P*<0.05); **(C)** glioma patients (*P*<0.05).

To explore the effect of β3GNT9 on glioblastoma patients’ survival, we evaluated the prognostic value of β3GNT9 using the “survival” package in R (**Figure 3A**). In addition, we also evaluated the prognostic value of β3GNT9 using the online database GEPIA. These Kaplan–Meier survival curves show high expression of β3GNT9 was associated with shorter OS (*P<*0.05, **Figures 3B, C**). In total, higher expression of β3GNT9 tends to have a worse prognosis compared to those with low expression.

**Figure 4:**
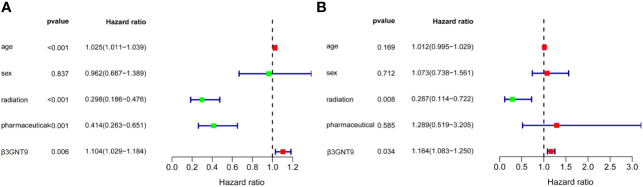
The results of univariate and multivariate COX regression analyses. **(A)** Univariate COX regression analysis of β3GNT9 expression and other clinical indicators. Age, radiation, pharmaceutical and β3GNT9 expression were indicators affecting prognosis. **(B)** Multifactorial COX regression analysis of β3GNT9 expression and other clinical indicators. Radiotherapy and β3GNT9 expression were independent factors affecting prognosis.

According to the univariate COX regression analysis (**Figure 4A**), age, radiotherapy, chemotherapy, and β3GNT9 were significantly associated with OS in glioblastoma patients (*P*<0.05). Incorporating the above variables and gender, the multivariate COX regression analysis showed that radiotherapy and β3GNT9 were independent prognostic indicators for patients. Radiotherapy is a protective prognostic indicator for glioblastoma (*P*<0.05), whereas high expression of β3GNT9 is an independent prognostic indicator for glioblastoma (**Figure 4B**).

### Gene enrichment analysis

**Figure 5:**
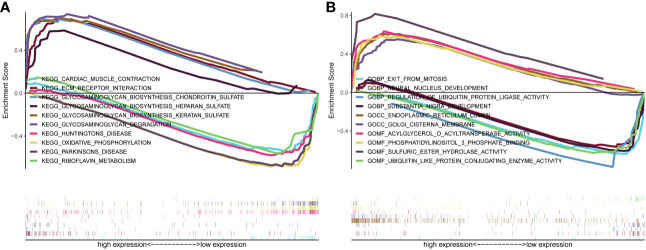
The result of gene enrichment analysis. **(A)** GSEA results showed differential enrichment of genes in KEGG with high and low β3GNT9 expressions. (The results showed ten significant KEGG pathways in the β3GNT9 high expression phenotype, including cardiac muscle contraction, ECM-receptor interaction, glycosaminoglycan biosynthesis chondroitin sulfate, glycosaminoglycan biosynthesis heparan sulfate, glycosaminoglycan biosynthesis keratan sulfate, glycosaminoglycan degradation, Huntington’s disease, oxidative phosphorylation, Parkinson’s disease, and riboflavin metabolism.) **(B)** GSEA results showed differential enrichment of genes in GO with high and low β3GNT9 expressions. (Five significant GO pathways in the β3GNT9 high expression phenotype included sulfuric ester hydrolase activity, acylglycerol-O-acyltransferase activity, phosphatidylinositol 3-phosphate bindin, endoplasmic reticulum lumen, and Golgi cisterna membrane. Five significant GO pathways in the β3GNT9 low expression phenotype included regulation of ubiquitin protein ligase activity, ubiquitin-like protein conjugating enzyme activity, exit from mitosis, neural nucleus development, and substantia nigra development).

Genes were subjected to GSEA analysis, and gene sets with *P*<0.05 and false discovery rate FDR<0.05 were considered as significantly enriched gene sets. The results showed ten significant Kyoto Encyclopedia of Genes and Genomes (KEGG) pathways in the β3GNT9 high expression phenotype, including cardiac muscle contraction, extracellular matrix receptor interaction, glycosaminoglycan biosynthesis chondroitin sulfate, glycosaminoglycan biosynthesis heparan sulfate, glycosaminoglycan biosynthesis keratan sulfate, glycosaminoglycan degradation, Huntington’s disease, oxidative phosphorylation, Parkinson’s disease, riboflavin metabolism (**Figure 5A**). Five significant GO pathways in the β3GNT9 high expression phenotype included sulfuric ester hydrolase activity, acylglycerol-O-acyltransferase activity, phosphatidylinositol 3-phosphate bindin, endoplasmic reticulum lumen, Golgi cisterna membrane (**Figure 5B**). Five significant GO pathways in the β3GNT9 low expression phenotype included regulation of ubiquitin protein ligase activity, ubiquitin-like protein conjugating enzyme activity, exit from mitosis, neural nucleus development, substantia nigra development (**Figure 5B**).

### Immune infiltration

**Figure 6:**
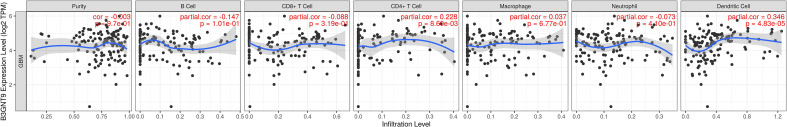
The expression of β3GNT9 was associated with immune infiltration in glioblastoma. β3GNT9 expression was positively associated with the infiltration levels of CD4+T cells, macrophages, dendritic cells (*P*<0.05), but negatively correlated with tumor purity, B cells, CD8+T cells, and neutrophils (*P*<0.05).

We investigated whether the expression of β3GNT9 was associated with immune infiltration in glioblastoma using the online database TIMER. The results revealed that β3GNT9 expression was significantly and positively associated with the infiltration levels of CD4+T cells, macrophages, dendritic cells (*P*<0.05), but negatively correlated with tumor purity, B cells, CD8+T cells, and neutrophils (*P*<0.05, **Figure 6**).

**Figure 7:**
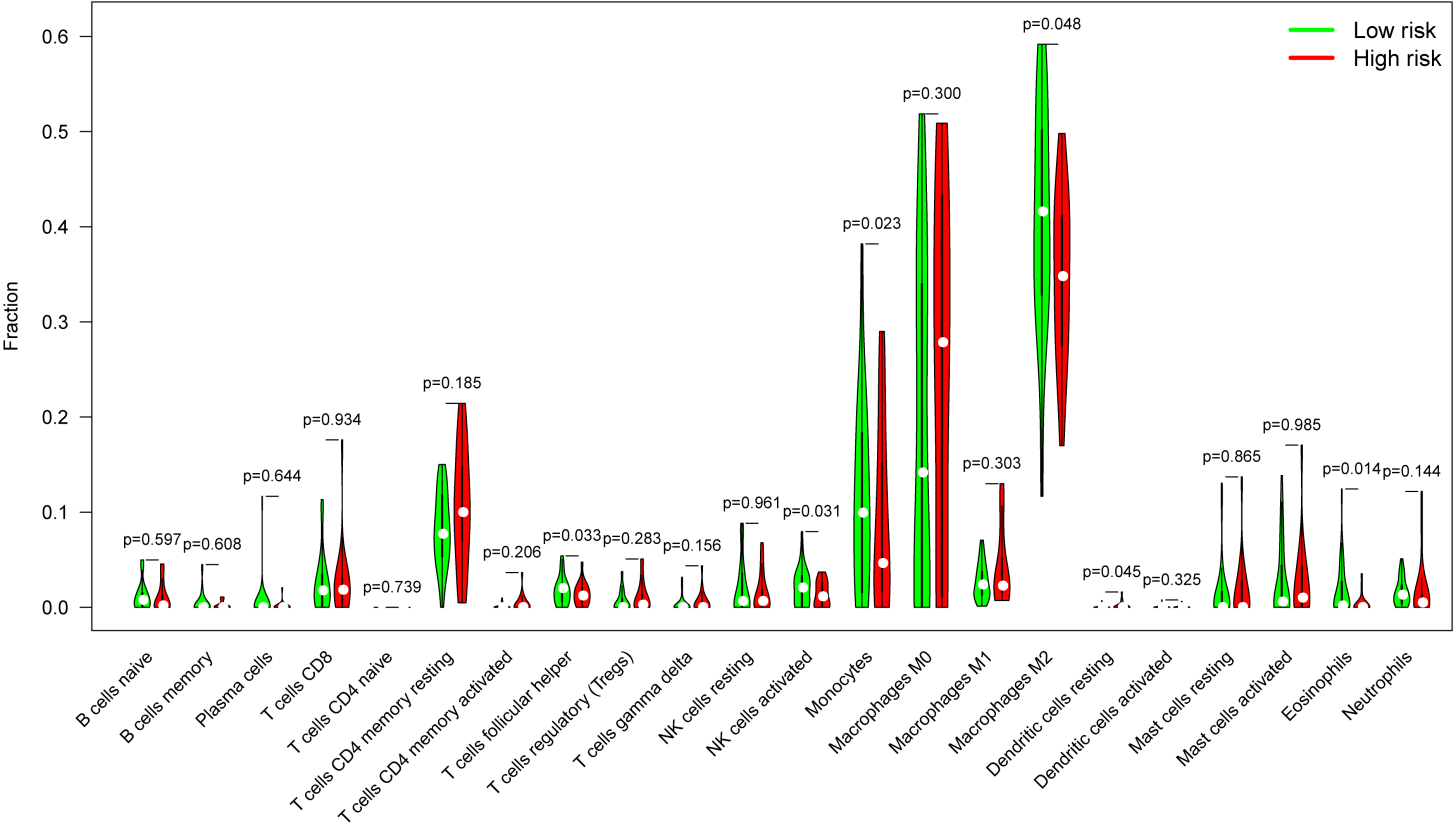
The proportion of 22 subpopulations of immune cells. T cells follicular helper, activated NK cells, monocytes, M2 macrophages, and eosinophils were the main immune cells affected by β3GNT9 expression. Among them and compared with the low expression group, helper follicular T cells (*P*=0.033), activated NK cells (*P*=0.031), mononuclear leukocytes (*P*=0.023), M2 macrophages (*P*=0.048), and eosinophils (*P*=0.014) in the high β3GNT9 expression group were significantly reduced.

**Figure 8:**
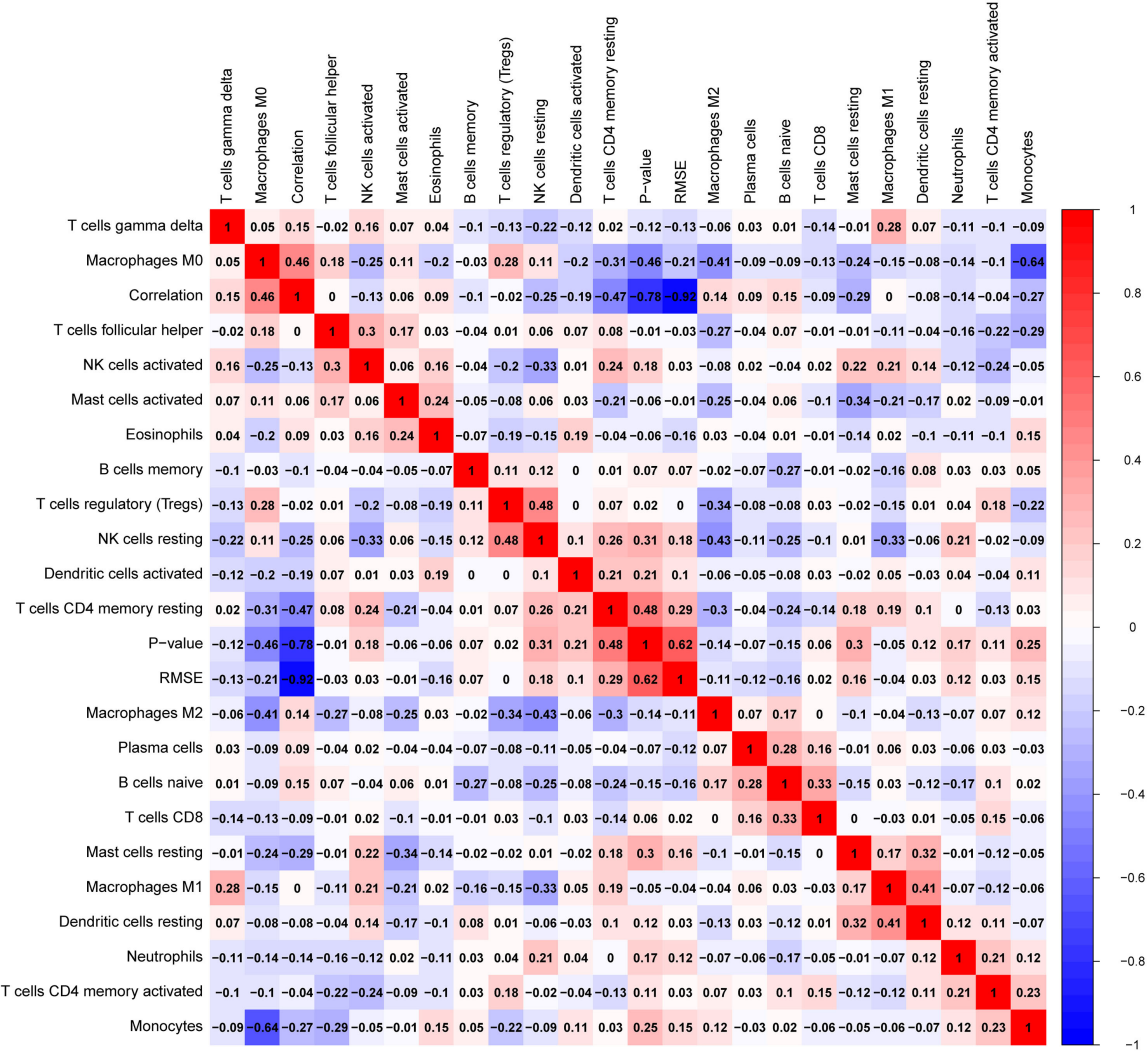
Heat map of 22 immune cells’ correlations in tumor tissue.

Glioblastomas were divided into β3GNT9 high expression half and low expression half, and the differences between the 22 immune cell expression levels were analyzed using the CBIERSORT algorithm. It was found that T cells follicular helper, activated NK cells, monocytes, M2 macrophages and eosinophils were the main immune cells affected by β3GNT9 expression. Among them and compared with the low expression group, helper follicular T cells (*P*=0.033), activated NK cells (*P*=0.031), mononuclear leukocytes (*P*=0.023), M2 macrophages (*P*=0.048), and eosinophils (*P*=0.014) in the high β3GNT9 expression group were significantly reduced (**Figure 7**). Afterwards, the heat maps of 22 immune cells were drawn as shown in **Figure 8**. Based on the immune infiltration analysis, we speculated that the high expression of β3GNT9 may lead to the change of activated NK cells and macrophages in tumor microenvironment, which in turn leads to poor prognosis of patients.

### β3GNT9 pan-cancer analysis

**Figure 9:**
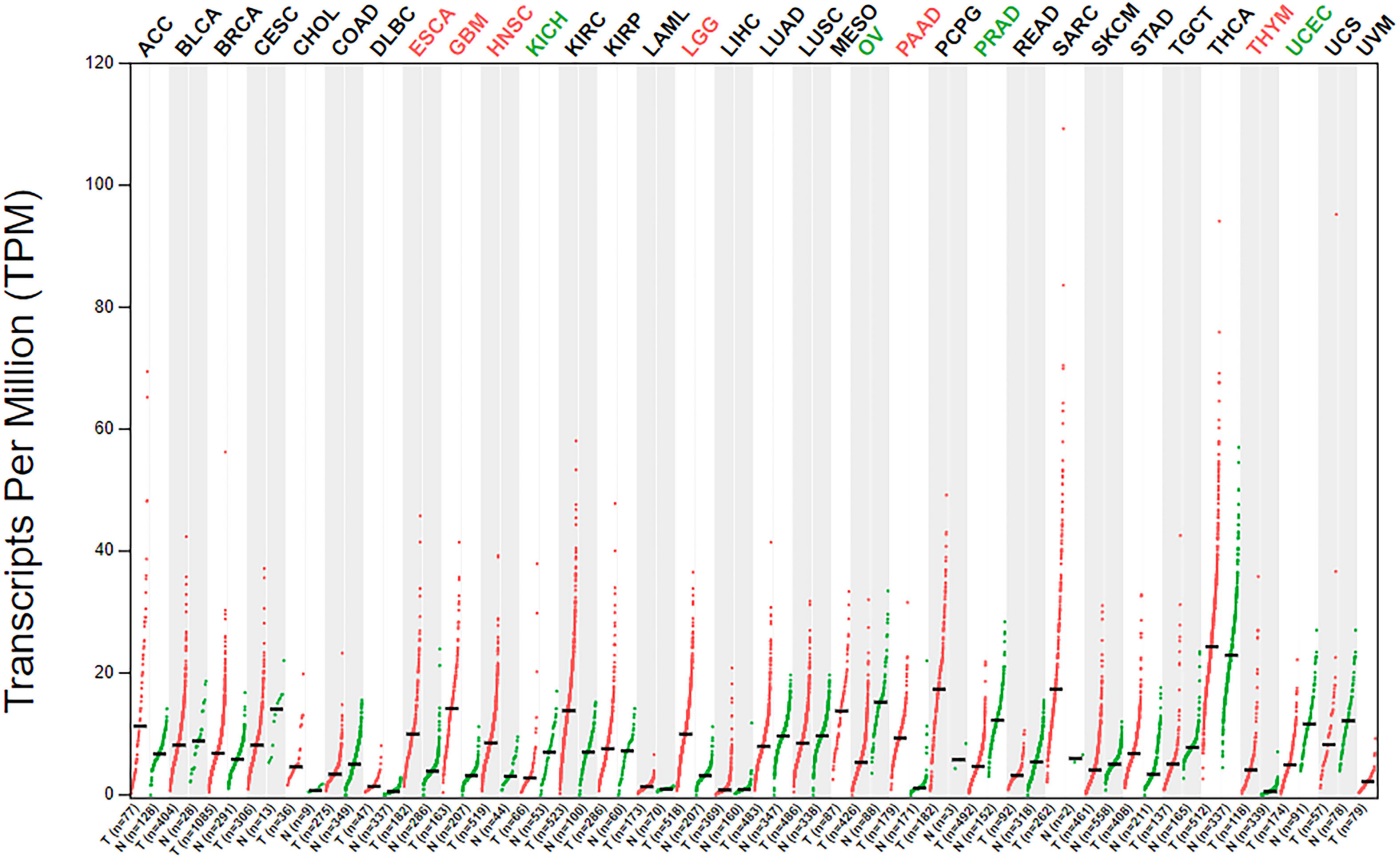
β3GNT9 expressions in 33 tumor tissues. β3GNT9 was high expressed in esophageal carcinoma, glioblastoma, head and neck squamous cell carcinoma, low-grade glioma, pancreatic carcinoma, and thymic carcinoma. In contrast, β3GNT9 was low expressed in kidney chromophobe, ovarian serous cystadenocarcinoma, prostate adenocarcinoma, and uterine corpus endometrial carcinoma.

The analysis of β3GNT9 expression in various tumors using the online database GEPIA revealed significant high β3GNT9 expression in esophageal cancer, glioblastoma, head and neck squamous cell carcinoma, low-grade glioma, pancreatic cancer, and thymic carcinoma (*P*<0.05), and significant low expression in chromophobe renal cell carcinoma, ovarian serous cystadenocarcinoma, prostate cancer, and endometrial cancer (*P*<0.05, **Figure 9**).

### Results of the qRT-PCR

**Figure 10:**
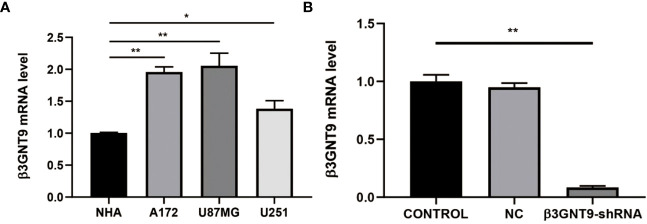
Detection of β3GNT9 expression in glioblastoma cell and NHA cell by qRT-PCR. **(A)** The β3GNT9 expression was higher in glioblastoma cell lines than in NHA cells (*P<0.05, **P<0.01). **(B)** mRNA levels declined after targeted β3GNT9 was silenced (**P<0.01). (* indicates *P*<0.05 compared with the control group; ** indicates *P*<0.01 compared with the control group).

We evaluated the expression of β3GNT9 in a variety of glioblastoma cell lines and normal astrocytes and found that β3GNT9 expression was higher in glioblastoma cell lines than in NHA cells (*P<0.05, **P<0.01, **Figure 10A**). The mRNA levels declined after targeted β3GNT9 silencing (**P<0.01, **Figure 10B**).

### Targeted β3GNT9 silencing significantly inhibited the migration and invasion ability of glioblastoma cells

**Figure 11:**
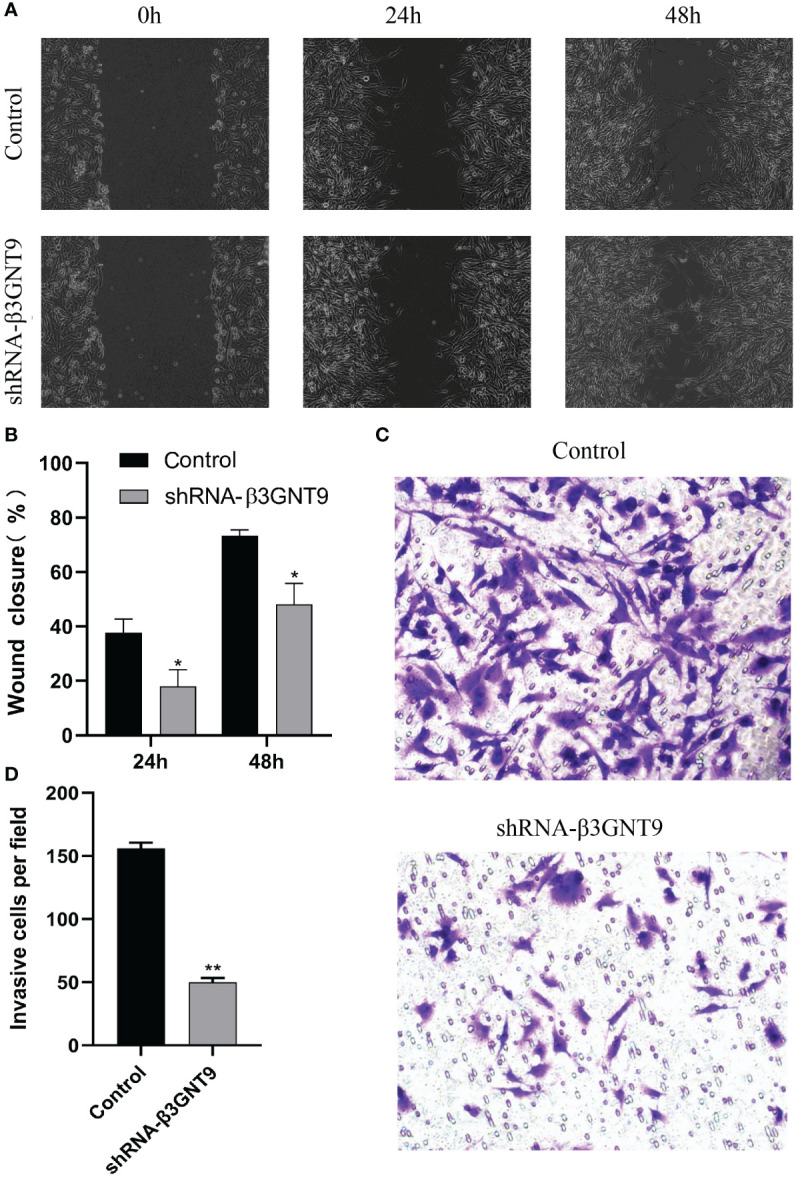
Targeted β3GNT9 silencing significantly inhibited the migration and invasion ability of glioblastoma cells. **(A, B)** The β3GNT9-shRNA group showed a much lower wound healing capacity than the control group (*P*<0.05). **(C, D)** The number of cells transfected by invading β3GNT9-shRNA was significantly lower than that of the control cells (*P*<0.01). (* indicates *P*<0.05 compared with the control group; ** indicates *P*<0.01 compared with the control group).

We performed cell scratch and invasion assays to examine whether the downregulation of β3GNT9 affected the invasion or migration ability of glioma cells. Cell migration was assessed using a scratch wound healing assay and the extent of cell migration to the scratch area was measured. The wounds in the wells of the control transfected cells healed rapidly, with almost no gaps left after 48 hours. However, the wells of β3GNT9-shRNA transfected cells showed a much lower healing capacity than the control wells (*P*<0.05, **Figures 11A, B**). Furthermore, the number of cells transfected by invading β3GNT9-shRNA was significantly lower than that of the control transfected cells (*P*<0.05, **Figures 11C, D**). These suggested that β3GNT9 silencing inhibited the migration and invasion capacity of glioblastoma cells.

## Discussion

The present study applied bioinformatics to analyze the molecular biological characteristics of human malignant glioma and found for the first time the high expression of β3GNT9 in glioblastoma, its correlation with prognosis, and its role in immune microenvironment. The findings indicate that increased β3GNT9 expression in glioblastoma can affect the immune microenvironment of glioblastoma and promote its migration and invasion. On this basis, we propose that β3GNT9 can be used as a potential independent prognostic biomarker for patients with glioblastoma. To the best of our knowledge, there has been no similar study reported in the existing literature, and we believe it will contribute to the evolution of anti-glioblastoma therapy.

β3GNT9 is a member of the β1-3-N-Acetylglucosaminyltransferases family (7). To date, many members of the β3GNTs family have been found to play a crucial role in the occurrence and progress of a variety of malignant tumors (8–10). For instance, research suggests that the suppression of the expression of β3GNT5 can reduce the formation of neurospheres, and glioblastoma patients with high β3GNT5 expression show explicitly reduced OS (23). In addition, the level of β3GNT8 increases with the increased pathological grade of gliomas, and high β3GNT8 expression catalyzes the migration of glioblastoma cells (24). Research also shows an obvious increase of β3GNT8 expression in invasive pituitary adenoma, and during the invasive growth of pituitary adenoma, β3GNT8, HG-CD147, and MMP-2 collectively increase the invasion of pituitary adenoma (25). β3GNT9 was used as the candidate gene for androgen-independent prostate cancer (26). However, there is no report on the biological function and mechanism of β3GNT9 in the progression of glioblastoma. To address this lacuna, we conducted the current research on the potential effect of β3GNT9 on the formation and development of glioblastoma. We made the first analysis of the high β3GNT9 expression in glioblastoma patients’ tissues, and found that it is significantly and negatively correlated to the prognosis of patients with glioblastoma. This finding has enriched the scholarly understanding of the role of the β3GNTs family in the occurrence and progression of central nervous system tumor.

Immunotherapy is a therapy that kills tumor cells by manipulating the autoimmune system (27). Previous studies have suggested that CNS is considered an immune-privileged organ because the brain lacks lymphatic cells and the blood-brain barrier for immune cells (28). However, recent research shows that the CNS has a certain degree of immune response (29). The unique immune microenvironment and structure of the CNS constitute a great challenge for the treatment of CNS tumors. Increasing evidence suggests that the tumor microenvironment influences tumor immune escape, immunotherapy response, and patient survival (30). Brain tumor immune microenvironment includes not only cytotoxic lymphocytes of the effective anti-tumor immune cells and NK cells, but also cancer-associated fibrocytes, endothelial cells, tumor-associated macrophages, among which macrophages are the most abundant (31). Tumor-associated macrophages infiltrate a large majority of solid tumors, promoting tumor progression by stimulating proliferation, angiogenesis, metastasis, and providing an anti-tumor immune barrier (32). Consistent with previous studies, the current study found β3GNT9 expression in glioblastoma is correlated negatively with tumor purity, but positively with macrophage enrichment. Besides, in glioblastoma, glioma stem cells secrete periostin, which recruits the tumor-promoting M2 subtype of macrophages into glioblastoma tissues, causing the malignant biological behavior of glioblastoma (33). Previous research reports that despite a relatively smaller proportion of NK cells in the infiltrate cell of glioblastoma, the small amount of NK cells in the glioblastoma immune microenvironment exhibit considerable cytotoxicity (34). Adding to previous research, our study found the higher the level of β3GNT9 expression, the fewer activated NK cells and follicular helper T cells. This means that a small amount of tumor infiltrating lymphocyte is not enough to induce powerful antitumor immune response and will cause immune evasion, consequently leading to poor glioblastoma patient prognosis. In this sense, β3GNT9 can be regarded as a potential novel target for glioblastoma immunotherapy.

Moreover, the results of the differential expression analysis of TCGA database combined with GTEx database revealed significantly increased β3GNT9 expression in glioblastoma tissues. Through qRT-PCR, we found the expression level of β3GNT9 was higher than that of normal astroglia in most cell lines of glioblastoma, which is in tune with the conclusion gleaned from relevant analysis using biological information technology. Afterwards, we used clinical information and RNA sequencing data to explore the relationship between β3GNT9 expression and the prognosis of patients with glioblastoma. Results showed they were negatively related. Clinical correlation analysis suggested that β3GNT9 expression was significantly higher in patients older than 65 years of age than in patients under 65 years of age. This finding is in line with previous studies concluding that β3GNT9 expression is associated with age (35). Furthermore, through univariate and multivariate COX regression analysis, we found the upregulation of β3GNT9 expression was an independent prognostic indicator of glioblastoma. Overall, our study has proved for the first time that high levels of β3GNT9 expression are related to the poor prognosis of glioblastoma and can serve as an indicator of glioblastoma.

In addition, through KEGG pathway analysis, we found potential relationship between β3GNT9 and the synthesis of glycosaminoglycan such as chondroitin sulfate, heparin sulfate, keratin sulfate, and so forth. This finding is consistent with existing research documenting that β3GNT7, which belongs to the same family as β3GNT9, may contribute to the biosynthesis of keratin sulfate (36). As such, we propose that β3GNT9 can promote glioblastoma cell proliferation through affecting the metabolism of glioblastoma.

Through the cell scratch invasion assays, this study also made the first attempt to find that the silencing of β3GNT9 constrained the migration and invasion of glioblastoma. Corroborating previous findings that β3GNT8, another member of the same family as β3GNT9, demonstrates a significant increase of expression in glioblastoma, and can fuel the proliferation, invasion, and migration of glioblastoma cell (23), our research also suggests that β3GNT9 may influence the migration and invasion of glioblastoma, which in turn, causes the malignant biological behavior of glioblastoma.

The present study has several limitations that can be used to suggest future directions. Firstly, we adopted primarily the bioinformatics approach and several vitro experiments. While we have gained preliminary understanding of the role of β3GNT9, future research should collect the tumor tissue samples of glioblastoma patients in the local hospital to further verify the impact of β3GNT9 expression on patients’ survival time. Secondly, confined by limited resources, more in-depth exploration of the molecular mechanism of β3GNT9 was not included in the present research, which tops the agenda of our follow-up work. Thirdly, some *in vitro* experiments in the present study used limited number of cell lines. Future research should use more cell lines to testify the results.

In conclusion, β3GNT9 demonstrates upregulated expression in glioblastoma, affecting the immune response of tumors and the immune microenvironment, especially macrophage and NK cells. High levels of β3GNT9 promote the migration and invasion of glioblastoma, which in turn leads to poor patient prognosis. Finally, high levels of β3GNT9 can serve as potential independent prognostic biomarkers for glioblastoma patients, promising a new direction of future immunotherapy for glioblastoma patients.

## Data availability statement

The datasets presented in this study can be found in online repositories. The names of the repository/repositories and accession number(s) can be found in the article/supplementary material.

## Ethics statement

Ethical approval was not required for the studies on humans in accordance with the local legislation and institutional requirements because only commercially available established cell lines were used. The manuscript presents research on animals that do not require ethical approval for their study.

## Author contributions

DJ, YL, and KW conceived and designed the project. YL, KW, LZ, and FZ performed the experiments. DJ, YL, FZ, and JZ analyzed the data. DJ, KW, YL, and SC drew the diagrams. DJ, YL, and XD wrote the manuscript. All authors contributed to the article and approved the submitted version.
